# The Role of Adolescents’ Personal and Social Resources in Achieving Desired Emotional and Behavioral Outcomes during an Anxiety-Provoking Pandemic Outbreak

**DOI:** 10.3390/ijerph18126280

**Published:** 2021-06-10

**Authors:** Shira Bukchin-Peles, Tammie Ronen

**Affiliations:** Department of Social Science, Tel-Aviv University, Tel-Aviv 69978, Israel; Tamie@tauex.tau.ac.il

**Keywords:** COVID-19, adolescents, positive personal resources, social resources, anxiety, handwashing

## Abstract

Considering adolescents’ developmentally driven stressors and social needs, they may be particularly vulnerable to the anxiety associated with the public health and economic crises due to the COVID-19 pandemic. Furthermore, they may have difficulty following the mandated contagion prevention directives. The current study focused on the role of adolescents’ positive personal resources (self-control, hope) and environmental resources (peer support) in two desired outcomes during the COVID-19 outbreak: wellbeing (i.e., maintaining/increasing positivity ratio) and contagion prevention behaviors (i.e., increasing handwashing). Path analysis was conducted using online survey data collected from a representative sample of 651 Israeli adolescents (ages 13–17). Positive resources were found to be both positively intercorrelated and negatively correlated with pandemic-related anxiety and positively with increased handwashing. Self-control correlated positively with social support, which, in turn, correlated positively with the positivity ratio (i.e., more positive than negative affects) and pandemic-related anxiety. Self-control and pandemic-related anxiety both correlated positively with increased prevention behavior. This study highlights the vital role of positive resources in achieving desired psychological and behavioral outcomes for adolescents during the anxiety-provoking pandemic. Beyond its theoretical innovation, this study offers practical value by focusing on malleable variables that could be the focus of dedicated interventions.

## 1. Introduction

During the highly contagious novel coronavirus disease (COVID-19) pandemic, the unique combination of the public health crisis, social isolation, and economic recession could have significant repercussions for adolescents’ wellbeing [1,2]. The COVID-19 outbreak has been accompanied by uncertainty and substantial personal risk, which have increased the public’s rates of anxiety, stress, and fear of the unknown. New studies have reported different psychological distress levels among people differently exposed to the COVID-19 pandemic [3,4,5], and adolescents may be particularly vulnerable. Furthermore, the World Health Organization [WHO] [6] has called for widespread public compliance with social distancing and hygiene measures, which may be especially challenging for adolescents. The current study focuses on paths to achieve the desired emotional and behavioral outcomes for adolescents to lessen the effects of the anxiety caused by COVID-19 and improve long-term capacities.

### 1.1. Why the COVID-19 Outbreak May Be Particularly Challenging for Adolescents

Adolescence is a significant transition period characterized by biological, behavioral, and psychological changes influenced by social conditions and family characteristics [7,8]. A wide range of cultural and environmental transformations occur during adolescence due to increased family responsibilities, academic and social demands, separation and individuation from the family unit, and exploration of stressful new experiences with peers and novel adult activities [9]. Moreover, executive functions, generally defined, include cognitive processes that underlie goal-directed behavior and are orchestrated by activity within specific areas in the brain [10]. These areas, related to executive functions, mature in adolescence, unlike many other brain regions that mature earlier. During this time, progressive (e.g., myelination) and regressive (e.g., synaptic pruning) changes occur concomitantly and are driven, in part, by an adolescent’s experiences, both internal and external [10]. These developments reinforce the emerging understanding of adolescence as a critical and sensitive period for the reorganization of regulatory systems [11]. Thus, this life stage is typically characterized by instability, which, in itself, may result in stress.

#### 1.1.1. Pandemic-Related Anxiety

The experience of living during COVID-19 likely adds further significant stressors to the lives of adolescents. In this rapidly changing situation, media and social conversations are entirely dominated by the outbreak. As a result, adolescents have had access to large amounts of information via social media, which can easily trigger stress [12]. They may fear becoming infected or worry about older significant others, such as their parents and grandparents, who could be at high risk of complications if infected. Furthermore, their everyday routines have been strikingly affected. At the time of the current data collection in mid-April 2020, governmental restrictions on movement and activity in Israel included closed schools and businesses, bans on social gatherings, guidelines to cease unessential work, and severe limitations on outings outside the home [13]. These, along with recommendations for the public to refrain from international travel and a 14-day mandatory quarantine for anyone returning from abroad or exposed to a confirmed COVID-19 case, transformed daily life and placed tens of thousands of Israeli adolescents in quarantine with their families.

**Figure 1:**
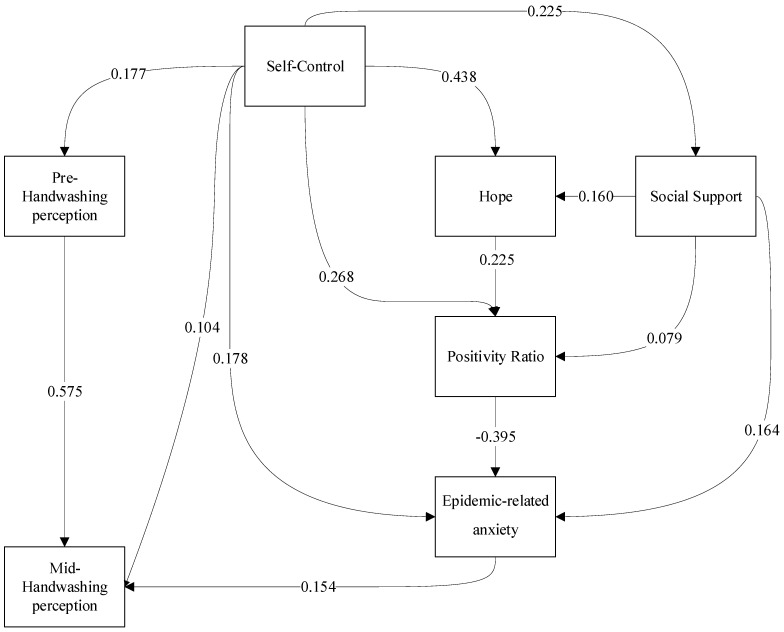
The mediation model. Note: Standardized regression weights are presented. All paths are significant.

Two main trends have characterized the study of anxiety. The environmental approach conceptualizes changes, anxiety, and stress as essential components that adversely influence one’s health [14]. The second approach focuses on typical development, asserting that humans respond “normally” even to severe crises. In extreme cases, anxiety may lead to indifference, “learned helplessness,” and even the inability to exercise self-control skills. However, studies suggest that, although anxiety may manifest as an increase in the frequency of behavioral problems, people do not usually develop post-traumatic stress disorder after exposure to trauma or stress. Moreover, after a time, they return to their usual behavioral patterns, relating to the event as a challenge [15,16]. Relatedly, exposure to the substantial stressors accompanying the COVID-19 outbreak has been shown to increase anxiety behavior levels [1,5,17,18,19,20]. As such, although excessive anxiety may decrease wellbeing, we suggest that some anxiety may be needed to prompt rapid behavioral changes in the short term, such as the recommended contagion prevention of handwashing. Thus, we expect that higher pandemic-related anxiety behavior will be directly linked to the desired increase in our study’s health promotion behavior—handwashing—during the COVID-19 outbreak (Figure 1).

#### 1.1.2. Handwashing

Alongside these measures, media announcements were disseminated to educate citizens on how to protect themselves and others from infection, thereby preventing and slowing down community transmission. At that time, people were instructed to wash their hands frequently or use an alcohol-based hand sanitizer, practice respiratory etiquette when coughing or sneezing (masks were not yet mandatory), refrain from touching their face, avoid close contact with people through social distancing, stay home, and self-isolate (WHO, [6]). In general, these extreme lifestyle transformations and directives, along with the fear of oneself or a loved one becoming ill, might have caused anxiety. However, this may have been especially true in the case of adolescents, who are already likely to be affected by a wide range of developmental, cultural, and environmental transformations.

Ideally, research should look into the actual social distancing measures as the desired behavioral outcome. However, this was not relevant during this survey due to the national lockdown. At the time of this study’s data collection, the WHO [6] indicated handwashing as the primary proactive and preventative action for individuals to protect themselves and others from the virus. Similarly, organizations and governments agreed that during the pandemic, one of the cheapest, easiest, and most important ways to prevent the spread of viruses was to wash hands frequently with soap and water (see review by [21]). Therefore, handwashing was the focus of the current study, as it was also particularly emphasized by the Israeli Ministry of Health.

### 1.2. Adolescents’ Positive Resources during the COVID-19 Outbreak

During this developmental stage, adolescents are regularly exposed to change, crises, and anxiety. Thus, empirical examinations of adolescents during the COVID-19 pandemic are critical to map out their resilient responses and positive resources for coping, despite their possible vulnerability. Can this sensitive population of adolescents flourish despite the anxiety-provoking situation? If so, what personal and social resources can enhance their resilience? Specifically, the current study focused on two desired positive outcomes during this period of anxiety and risk: (1) wellbeing through maintaining or increasing one’s positivity ratio (having more positive than negative affects) despite the pandemic; and (2) increasing one’s contagion prevention behavior (handwashing). To better understand how to achieve these two psychological and behavioral outcomes, we chose to investigate the role of personal and social resources; namely, self-control and hope denoted positive personal components, and peer social support denoted the positive environmental component. In a previous investigation, positive components were empirically associated with these desired outcomes in an adult sample during the pandemic outbreak [22]. Thus, the current study sought to determine how this might also relate to adolescents coping with the COVID-19 outbreak.

We propose a mediation model of personal and environmental resources for promoting adolescents’ wellbeing and behavioral change during the pandemic outbreak. The model’s mediating variables were chosen based on previous studies that linked self-control to lower rates of anxiety [15,23,24,25], increased hope [26], and adolescents’ ability to build their social support system [27,28]. Thus, altogether, we expect that higher self-control skills will be positively associated with adolescents’ higher positivity ratio, increased contagion prevention behavior (handwashing), stronger hope, reduced anxiety, and more significant social support, despite the stressful situation (Figure 1). The following sections will elaborate on the different variables that compose the suggested model.

#### 1.2.1. Positivity Ratio

In the current study’s mediation model, we used the concept of the positivity ratio to denote wellbeing. The positivity ratio conceptualization derives from the idea that positive emotions and negative emotions operate as independent bipolar constructs. The existence of one does not necessarily point to a lack of the other [29,30,31,32]. According to Fredrickson [33], a ratio of positive to negative emotions of approximately 3:1 is the ratio at which the dynamic structure bifurcates between a limited cycle of languishing and the complex dynamics of flourishing. Gottman [34] suggested the ratio should be about 5:1 (positive to negative). However, recently, the existence of a specific ideal ratio has been generally questioned [35].

Positive emotions include pleasant or desirable situational responses, ranging from interest and contentment to love and joy. Positive affect is considered a marker of overall wellbeing or happiness [36,37]. In addition, experiencing positive emotions is also associated with better functioning and, in the long run, with enhanced physical, intellectual, and social resources [38] and future growth and success [39]. Positive emotions are, therefore, a crucial component for achieving resilience. Several popular measures quantify positive emotions (e.g., [30,40,41]).

Everyone experiences negative emotions, but wellbeing can be determined from the extent to which one experiences more positive affects than negative affects [32,33,42]. The ability to have more positive than negative emotions may be especially important for adolescents, considering their characteristic age-related changes and reorganization of regulatory systems [11]. Moreover, in times of crisis and uncertainty, like in the current pandemic, maintaining a high positivity ratio may be more critical than usual. Previous research has highlighted self-control and hope (personal resources) as well as social support (environmental resources) as possibly facilitating a higher positivity ratio in times of crisis [27,28]. In the current mediation model, we investigated positive resources’ role in relation to adolescents’ positivity ratio as a proxy for wellbeing.

#### 1.2.2. Self-Control

We conceptualized self-control skills as playing a central endogenous role in the current mediation model. Self-control is a set of skills that begins to evolve from birth and gradually increases throughout one’s development. As it evolves, it enables the development of other skills such as learning, experiencing emotions, and occupational and social competencies [43]. Such skills enable people to work toward their goals, postpone gratification, and overcome difficulties relating to thoughts, emotions, and behaviors (Rosenbaum, [44]). Thus, adolescents are in a stage where they have already attained a certain level of self-control skills; however, their goal-directed skills are not yet complete.

Self-control may be of particular relevance in coping with the COVID-19 outbreak due to the goal-directed “redressive” behavior (Rosenbaum, 1980), which helps people overcome stressful situations, pain, and disturbing emotions [45,46,47,48]. Rosenbaum [49] defined self-control as the process by which individuals consciously decide to take charge of their behavior, especially when automatic and habitual responses have been interrupted or found ineffective. For example, research has suggested that self-control is a crucial personal component in coping with stressful war situations [15,25,50] {Rosenbaum, 1991 #1006. Importantly, self-control targets both internal and external disturbing conditions [15,25].

Self-control was conceived in this study as an essential personal skill that influenced the positivity ratio, perceived handwashing behavior, hope, pandemic-related anxiety, and social support. Studies informing our model’s desired psychological outcome of the positivity ratio found that self-control skills were linked to the ability to attain wellbeing, happiness [24,43,49,51,52], hope [53,54], social support [27,55], and positive emotions [27,28,55,56]. Regarding our model’s desired behavioral outcome of handwashing, a study by Rosenbaum [49] maintained that self-control plays a “reformative” function by facilitating the adoption of new types of behaviors that require delaying gratification, resisting temptations, and developing new habits. This would be relevant in adopting the recommended handwashing behaviors.

#### 1.2.3. Hope

Snyder [57] defined hope as the perceived ability to derive pathways to desired goals and motivate oneself through agency thinking, which is the belief in bringing about change and targeting a specific goal. This definition has been the most commonly used and analyzed in recent times [58,59]. Snyder outlined hope as a dynamic cognitive, motivational system that helps people look toward a better future, enhances coping with difficulties (e.g., sickness, trauma, and disaster), and serves as a critical component in attaining wellbeing [60,61,62,63].

Following theoretical arguments related to Snyder’s [64] hope theory, our mediation model posited that hope would influence the positivity ratio and mediate the relation between self-control and the positivity ratio. Studies have supported the importance of hope among adults, demonstrating that high levels of hope serve as a buffer against stress and trauma and are associated with higher wellbeing and numerous positive outcomes [64,65,66,67,68,69]. In health psychology, studies have revealed that people with high levels of hope exhibit more constructive thinking when problem solving [70]. Hope was also found to be associated with life satisfaction [70,71] and mediate the negative potential of fear [72]. Given their ability to think constructively and overcome negative emotions such as fear, people with higher levels of hope may also reveal a higher positivity ratio (i.e., experiencing more positive than negative emotions). Therefore, hope may be an essential positive cognitive resource for adolescents as they experience mood changes and focus on the near future and their habitual way of living. Such a resource could enable them to look toward a better future. Thus, not only does our mediation model conceive of hope as associated with a higher positivity ratio but we also conceptualize the positive outcome of increased hope as important in and of itself.

With regard to handwashing, hope has been significantly linked to taking action and problem solving [64], especially in situations where one believes the chances of success are limited [73]. Studies have suggested that higher levels of personal hope promote the anticipation of a better future. Thus, more hopeful people are encouraged to set more complicated and ambitious goals and take action [61,64,73]. Accordingly, during a pandemic outbreak, when people face uncertainty, fear, and actual risk, more hopeful people may attempt to take further action to increase the chances of staying safe. We suggest that more hopeful adolescents may be more likely to wash their hands in more situations, even if they are unsure of the odds of success.

#### 1.2.4. Peer Social Support

Sarason, Pierce [73] established that social support is an environmental coping resource. Adolescence is characterized by the modification and expansion of social ties [74]. As such, at this stage of life, the role of social support in wellbeing is emphasized, encompassing the importance of emotional support, understanding, intimacy, and loyalty [75,76]. Furthermore, it is relevant in effectively overcoming difficulties and maintaining subjective wellbeing [24,75,77,78].

Peer support has been shown to contribute substantially to adolescents’ positive functioning [79,80]. Adolescents who perceived receiving social support from multiple sources have demonstrated more positive school outcomes such as attendance, engagement, grades, and school satisfaction (Rosenfeld, Richman, and Bowen, 2000). They were also shown to attain higher levels of subjective wellbeing, mental health, physical health, and longevity [74,81]. Perceived peer support was also found to correlate with adolescents’ lower rates of depression [82] and has been seen as a crucial resource for helping adolescents cope with fear [24]. However, the COVID-19 outbreak necessitates adolescents, who gain numerous positive outcomes from social support, to stay home and socially distance themselves, thereby cutting themselves off physically from one of their primary sources of support.

Adolescents are particularly vulnerable to peer influence for several reasons, such as looking to their friends to understand social norms. Consequently, over time, they may align their behavior with their social group’s norms or the group they want to belong to [83]. Moreover, adolescents may find it particularly rewarding to gain social status, a potential outcome of aligning with peers. Hence, peers may also negatively influence one another, for example, by increasing the likelihood that adolescents will take certain risks [19,84]. Finally, adolescents tend to be hypersensitive to the adverse effects of social exclusion. Thus, the desire to avoid the risk of being ostracized may outweigh the potential negative consequences associated with health risks or illegal behaviors [85].

In relation to COVID-19, adolescents have been conceived of as a possibly pronounced source of community contagion as they may find it challenging to comply with social distancing and hygiene directives. This is likely due to their age-appropriate need for peer engagement and individuation from their nuclear family, along with increased risk taking [19,84] and their sense of invulnerability, especially in light of the media attention given to the lower mortality and disease severity rates of COVID-19 for younger persons [86]. Adolescents are highly influenced by their friends, more so than adults [87,88,89]. Thus, for adolescent groups engaging in high-risk behaviors or whose social norms dictate that governmental health directives (i.e., handwashing and social distancing) are “only for old or sick people”, the desire to avoid ostracization may outweigh the potential negative consequences associated with the health risk [85]. Prior interventions and campaigns aimed at influencing adolescent behavior have often been unsuccessful [90]. Furthermore, considering the scarcity of available evidence on anxiety or difficulties among adolescents during the COVID-19 outbreak, we expect the current study’s findings to hold implications for policymakers, educators, parents, and health workers.

In addition, due to the strong influence of adolescents on one other, we suggest that close relations with peers who are highly stressed by the COVID-19 outbreak may increase anxiety within the peer group. Thus, we hypothesize that peer support will positively correlate with positive components such as hope and the positivity ratio. Nevertheless, the relation between social support and COVID-19-related anxiety depends on peers’ feelings as well. For example, if an adolescent’s friends are more anxious about the pandemic, this may contribute to their anxiety. We will explore this through questions relating to adolescents’ perceptions of their friends’ ability to encourage and increase either positive or negative activities, which may be affected by their anxiety.

Thus, the current study focuses on two desired positive outcomes during a pandemic outbreak: (1) maintaining or increasing one’s positivity ratio despite the pandemic, and (2) increasing one’s contagion prevention behavior. To better understand how to attain these two psychological and behavioral outcomes, we will explore the role of positive personal (i.e., self-control and hope) and environmental (i.e., peer social support) resources.

## 2. Method

### 2.1. Sample and Procedure

The survey was conducted over one week during the COVID-19 pandemic outbreak in Israel (14–21 April 2020) among 651 teenagers aged 13–17 years old (*M* = 15.5, *Mdn* = 16.0, *SD* = 1.35), with 56% being females (*SD* = 0.49). The professional online panel service Panel4All (available online: http://www.panel4all.co.il/ accessed on 20 April 2021) recruited a representative sample of the adolescent Israeli population while taking into consideration sex, age, and residential area. All participants gave informed consent to participate in the study after being guaranteed anonymity. The Tel Aviv University Institutional Review Board gave ethical approval for the study.

At the time the online survey period began (14 April), Israel had reported 11,868 confirmed cases of COVID-19, of whom 181 were in severe condition, with 118 deaths, while the global death toll moved past 120,000. By the end of the sampling period (21 April), the reported number of confirmed cases had risen to 13,883, with 142 people in severe condition and 181 deaths. During the survey, schools, public buildings, and most businesses had been shut down, and government directives had been issued instructing citizens only to leave their homes to buy food and medication. The majority of Israelis complied with the governmental directives. During the first few days of the sampling period (14–16 April), the police strictly enforced a severe nationwide lockdown. Intercity roadblocks were set up to prevent Israelis from attending extended family gatherings celebrating the end of the Passover holiday. On 19 April, steps to ease the shutdowns were announced, and the new guidelines permitted a return to work for those in specific industries, reopening of stores, and more. Throughout the sampling period, only 15% of workers were deemed essential and permitted to go to work; later, 30% were classified as essential. The 25% unemployment rate remained constant during the sampling period.

### 2.2. Measures

The online survey included six self-report measures and socio-demographic information on age, sex, grade level, household size, and residential area.

#### 2.2.1. Self-Control Skills

The 32-item youth version of the Self-Control Scale [51] assesses self-control skills, including problem-solving skills, attention control/distraction, cognitive reframing, delay of gratification, and use of self-talk and self-reinforcement. Participants rated items (e.g., “When I am bothered by unpleasant thoughts, I try to think about more pleasant things”, “When I need to make a decision, I try to weigh all the possible options in order not to act impulsively”) on a scale ranging from −3 (not characteristic of me at all) to +3 (very characteristic of me). Responses to all items were summed (while subtracting reverse-coded items that were negatively worded). Higher scores indicated higher self-control skills. This scale has been previously used in the Israeli context (see, e.g., [27,55,91]). In the present study, the Self-Control Scale’s Cronbach alpha was 0.76.

#### 2.2.2. Positivity Ratio

The 20-item Positive and Negative Affect Schedule [30] is a self-report checklist of adjectives assessing independent measures of positive affect (e.g., excited, proud) and negative affect (e.g., upset, guilty), with ten items each. Participants rated the frequency they experienced the 20 emotions in the last week on a scale ranging from 1 (never) to 5 (always). In the current study, internal consistency was α = 0.82 for positive emotions and α = 0.82 for negative emotions. The mean score for the positive affect subscale was divided by the mean score for the negative affect subscale to calculate the positivity ratio. A larger ratio of positive to negative emotions (higher score) represented a higher positivity ratio, thereby indicating greater wellbeing. This scale has been previously used in the Israeli context (see, e.g., [55,91]).

#### 2.2.3. Perception of Handwashing (before and during the Outbreak)

A handwashing checklist was presented twice to participants, once regarding handwashing at the current time of the pandemic outbreak and once retrospectively regarding their routine handwashing behavior before the outbreak. We asked the participants to mark all circumstances in which they currently/previously washed their hands: before every meal; after every meal; during food preparation; before and after wound treatment; after blowing my nose, coughing, or sneezing; after touching trash; after using the toilet; during my travels; after meeting people in public places; when I arrive home; after any contact with an animal or with animal feces; after changing diapers or cleaning a child who has used the toilet. The list of circumstances was taken from the State of Israel Ministry of Health’s online handwashing recommendations. Participants were asked to mark all applicable answers. For the analysis, we summed each participant’s number of marked answers (e.g., a score of 6, if six handwashing circumstances were marked) to designate participants’ perceptions of their own handwashing behavior.

#### 2.2.4. Hope

The 8-item Hope Scale [63], also known as the Goals Scale, is a cognitive, goal-oriented measure assessing four agency items (e.g., “My past experiences have prepared me well for my future”) and four pathway items (e.g., “I can think of many ways to get out of a jam”), interspersed with four filler items (distractors intended to make the scale content less obvious) not included in the scoring. Prior studies revealed the scale’s sound internal reliability, with Cronbach alphas ranging from 0.74 to 0.88 [61,92,93]. Its convergent validity has been substantiated by its predicted correlations with several other scales designed to measure similar concepts [94]. This scale has been previously used in the Israeli context (see, e.g., [91,95,96]). In the present study, the Hope Scale’s Cronbach alpha was 0.83.

#### 2.2.5. Pandemic-Related Anxiety

A novel 10-item questionnaire was developed for this study to assess stress reactions to the pandemic. Participants rated items (e.g., “I am very concerned about the coronavirus outbreak”, “I worry that my family will get sick”, and “It is hard for me to sleep because of my worrying about the coronavirus”) on a scale ranging from 1 (never) to 6 (always). Responses to all items were summed (while subtracting reverse-coded items that were negatively worded). Higher scores indicated higher anxiety related to the pandemic. As this is a new instrument, Pearson’s correlation was employed to assess the construct validity [97]. The correlation matrix analysis revealed that all ten questions presented a positive correlation, with a significance level lower than 0.01 (see Table S1 in the Supplementary Material). The Cronbach alpha for the pandemic-related anxiety scale was 0.85.

#### 2.2.6. Peer Social Support

The 12-item peer social support subscale of the self-reported Child and Adolescent Social Support Scale assesses the perceived frequency of social support by peers (Malecki, 2002). Participants rated items (e.g., “My peers give me good advice”, “My friends comfort me”) on a scale ranging from 1 (never) to 6 (always). Reliability for the peer social support subscale was α = 0.94 in a previous study conducted in Israel (Orkibi, 2018) and 0.94 in the current study.

### 2.3. Data Analysis

After examining current and prior handwashing self-reports using *t*-tests for paired samples, path analysis was conducted to test the hypothesized mediation model. The model’s fit to the data was evaluated using the criteria of χ^2^/df ≤ 3, comparative fit index (CFI) ≥ 0.95, Tucker–Lewis coefficient (TLI) ≥ 0.95, and root mean square error of approximation (RMSEA) < 0.80 [98]. The bootstrap method was utilized to test for indirect effects (i.e., mediation), with the confidence level set at 0.95 and bootstrap bias-corrected samples set at 5000. When zero is not in the 95% confidence interval (CI), the indirect effect is significantly different from zero at two-tailed *p* < 0.05 [99].

## 3. Results

### 3.1. Preliminary Analyses

Table 1 displays the means, standard deviations, and maximum and minimum values for the six study variables. As seen in the descriptive statistics in the table, a paired sample *t*-test was conducted to compare perceived handwashing behavior before the epidemic versus during the epidemic, yielding a significant difference, *t* (651) = 21.301, *p* < 0.01 (2-tailed). This finding suggests that the coronavirus outbreak significantly increased adolescents’ reported number of everyday handwashing circumstances (e.g., when returning home, after eating, after meeting people, rather than only after using the bathroom). Thus, pre-epidemic handwashing behavior was included in the path analysis.

As the theorized mediated model began with self-control as the independent variable, we examined its possible correlations with adolescents’ age and sex. A higher self-control level was associated with older age (B = 0.117, SE = 0.029, *p* < 0.001) and female sex (calculated where male = 1: B = −0.178, SE = 0.078, *p* = 0.22). These findings coincide with the existing literature. Including sex and age in the analysis did not affect the other associations found.

### 3.2. Path Analysis

The path analysis indicated that the theorized model depicted in Figure 1 provided a good fit to the data on the following fit indices: χ^2^/df = 2.08, CFI = 0.989, TLI = 0.972, RMSEA = 0.041. Table 2 displays the estimated regression weights, indicating a series of direct effects (see Figure 1).

The complete mediation model is displayed in Figure 1. In addition to the direct effects shown in Table 2 and Figure 1, several significant indirect, mediated effects were found. The mediated effect of self-control on hope was 0.63, in addition to the direct effect (B = 0.438, SE = 0.035, *p* < 0.001). Bias-corrected bootstrap analysis of indirect effects indicated a significant indirect association between self-control and hope (95% CI, *p* < 0.001, two-tailed). Thus, social support mediated the relation between self-control and hope. The mediated effect of self-control on the positivity ratio was 0.124, in addition to the direct effect (B = 0.268, SE = 0.04, *p* < 0.001). Bias-corrected bootstrap analysis of this indirect effect indicated a significant indirect association (95% CI, *p* < 0.001, two-tailed). Social support and hope mediated the relation between self-control and the positivity ratio. The mediated effect of self-control on pandemic-related anxiety was −0.118. This is in addition to the direct effect (B = 0.178, SE = 0.04, *p* < 0.001). Bias-corrected bootstrap analysis of this indirect effect indicated a significant indirect association (95% CI, *p* < 0.001, two-tailed). Social support, the positivity ratio, and hope mediated the relation between self-control and pandemic-related anxiety. The mediated effect of self-control on handwashing during the pandemic was 0.111, in addition to the direct effect (B = 0.140, SE = 0.031, *p* < 0.001). Bias-corrected bootstrap analysis of this indirect effect indicated a significant indirect association (95% CI, *p* < 0.001, two-tailed). Social support, the positivity ratio, hope, handwashing prior to the pandemic, and pandemic-related anxiety mediated the self-control and handwashing during the pandemic relation.

Overall, higher self-control was positively (directly and indirectly) related to increased handwashing habits during the pandemic outbreak. At the same time, higher self-control was positively (directly and indirectly) linked to positive components (hope and positivity ratio). Furthermore, self-control was positively related to social support and negatively (indirectly) linked to pandemic-related anxiety.

The other positive components in this model (i.e., hope and positivity ratio) were also significantly indirectly associated. The mediated effect of hope on pandemic-related anxiety was −0.089. Bias-corrected bootstrap analysis of this indirect effect indicated a significant indirect association (95% CI, *p* = 0.001, two-tailed). The relation between hope and pandemic-related anxiety was mediated via the positivity ratio. The mediated effect of hope on handwashing during the pandemic was −0.014. Bias-corrected bootstrap analysis of this indirect effect indicated a significant indirect association (95% CI, *p* = 0.001, two-tailed). The relation between hope and handwashing during the pandemic was mediated through the positivity ratio and pandemic-related anxiety. The mediated effect of the positivity ratio on handwashing during the pandemic was −0.061. Bias-corrected bootstrap analysis of this indirect effect indicated a significant indirect association (95% CI, *p* = 0.001, two-tailed). The relation between the positivity ratio and handwashing during the pandemic was mediated by pandemic-related anxiety. Therefore, positive components were, directly and indirectly, related to decreased pandemic-related anxiety and increased handwashing habits during the pandemic outbreak.

Social support also indirectly affected several variables. The mediated effect of social support on the positivity ratio was 0.036, in addition to the direct effect (B = 0.079, SE = 0.036, *p* = 0.03). Bias-corrected bootstrap analysis of this indirect effect indicated a significant indirect association (95% CI, *p* < 0.001, two-tailed). The relation between social support and the positivity ratio was, thus, mediated by hope. The mediated effect of social support on pandemic-related anxiety was −0.045, in addition to the direct effect (B = 0.164, SE = 0.037, *p* < 0.001). Bias-corrected bootstrap analysis of this indirect effect indicated a significant indirect association (95% CI, *p* = 0.001, two-tailed). The positivity ratio and hope mediated the relation between social support and pandemic-related anxiety. Social support indirectly affected handwashing during the pandemic. Hope, the positivity ratio, and pandemic-related anxiety mediated the relation between social support and handwashing during the pandemic. The mediated effect of social support on handwashing during the pandemic was 0.018. Bias-corrected bootstrap analysis of this indirect effect indicated a significant indirect association (95% CI, *p* = 0.002, two-tailed). Hence, social support was directly and indirectly associated with positive components (hope and positivity ratio) and increased handwashing habits during the pandemic outbreak and increased pandemic-related anxiety.

## 4. Discussion

Uncertainty about the personal and global effects of the COVID-19 outbreak, as well as the specific effects of quarantine and social isolation, is a source of great concern in general and specifically for educators. Adolescents may be at exceptionally high risk of experiencing coronavirus-related anxiety and not adhering to health guidelines and recommendations. This study aimed to identify possible positive resources that may help design timely actions to minimize adverse effects and improve young people’s long-term capacities.

The current findings indicate that higher self-control was positively related to the two desired health outcomes: a greater positivity ratio and increased handwashing behavior during the pandemic outbreak. At the same time, higher self-control was positively linked with hope and peer social support and negatively linked with pandemic-related anxiety.

This study highlights the importance of positive personal components for human coping, particularly in times of global crisis. Positive components were found to be related to decreased pandemic-related anxiety and increased handwashing behavior during the outbreak. These findings coincide with prior research, which indicated that self-control, positive emotions, and hope are associated with several positive outcomes related to health, success, and wellbeing (see reviews by [64,100,101,102]).

Past research on self-control usually focused on its primary role in decreasing various types of undesired behaviors [24,25,103]. However, the current results also highlight self-control’s constructive role in increasing desired routine behaviors, such as washing hands (e.g., [103]) and promoting desired mental and physical health indices. Therefore, these findings expand on prior research by indicating self-control’s link to increases in hope and positive emotions (Orkibi et al., 2018; Rosenbaum and Ronen, 2013).

The current outcomes appear to underscore the possible multifaceted role of adolescents’ skills in following instructions, coping with demands, delaying temptations (i.e., self-control), and their ability to focus on hope and positive emotions despite the stressful situation inherent to the epidemic outbreak. Regarding psychological health, such self-control skills may foster greater hope regarding their sense of agency and specific pathways toward a better future. This may then promote the capacity to maintain a higher ratio of positive to negative emotions, indicating better wellbeing. A higher positivity ratio may, for example, help people broaden their repertoire and creatively find ways to overcome the social distancing regulations (e.g., initiating online video conversations, setting up outdoor meeting venues). As a lower positivity ratio is negatively linked to pandemic-related anxiety, the current finding substantiates prior research, indicating that a higher positivity ratio is related to wellbeing, coping abilities, and less anxiety [33,104,105].

Regarding increases in contagion-preventing hygiene behaviors, the current finding coincides with previous research showing that people experiencing high anxiety try to find a solution for the root cause of their anxious behavior. Further research should explore whether handwashing behavior increases in a rational manner during a pandemic outbreak to prevent infection as recommended by the authorities or if it becomes an irrational, over-washing compulsion.

The current findings regarding social support merit special consideration in light of the heightened role of peer relations during adolescence. Social support was associated with positive components (i.e., hope and positivity ratio) and increased handwashing behavior during the pandemic outbreak. Yet, higher social support was also found to be linked to an increase in pandemic-related anxiety. This is somewhat surprising as social support could be assumed to ameliorate the higher anxiety that might be expected due to the substantial changes adolescents are experiencing during the pandemic to their daily social routines and social infrastructures, which ordinarily help foster adolescents’ resilience to challenging events [5]. However, the current finding may be related to how adolescents see their level of anxiety regarding the pandemic compared to their peers. In this study, on a scale of 1 to 6 for the pandemic-related anxiety scale item “I am more anxious about the coronavirus outbreak than my peers,” the mean score was only 2.39 (*SD* = 1.44), suggesting that the participants saw their peers as relatively more anxious than themselves. If this is the case, peers may reflect their anxious thoughts on each other, causing an increase in pandemic-related anxiety levels.

## 5. Conclusions

In addition to its theoretical innovation, this study’s importance lies in its practical value. The variables examined herein are malleable and could be influenced through dedicated interventions and curriculums. Although COVID-19 generally appears to pose a low physical risk to adolescents themselves, it may affect their wellbeing and willingness to follow contagion-reducing guidelines, which are essential to reducing the risk for others. Hence, policies, interventions, and cultural practices aimed at strengthening personal resources are expected to boost the welfare of the population as a whole, including adolescents [106].

Various interventions have been shown to increase personal resources and, specifically, self-control (e.g., [66,106,107,108,109,110,111]). Future research should continue to investigate the precise types of interventions that may encourage the desired outcomes in the time of a pandemic. The current perspective also suggests that educators, who have a critical role in managing the COVID-19 outbreak, could be at the core of future interventions to address positive personal and social resources. Such interventions may also include support groups and evidence-based use of different media tools.

Limitations, and Directions for Future Research. Future studies should also consider the family and social context of participants (i.e., number of family members, personal contact with infected individuals, personal positive COVID-19 test, familiarity with COVID-19 casualties). These variables may be linked to other variables in the study, specifically, pandemic-related anxiety.

Moreover, future research should deepen the methodological analysis for the new pandemic-related anxiety construct. This instrument should be validated against other new instruments invented at the time of the pandemic. Content validity, criterion-related validity, and construct validity should all be evaluated. Although we confirmed the reliability of the construct, other measures could also be used to further evaluate this aspect (e.g., ω coefficients, greatest lower bound (GLB)).

The association between positive resources and management of a pandemic outbreak should be further explored to include other types of pandemics and epidemics in different communities and ages, as well as other positive components. Future studies should also rely on a larger and more diverse sample and collect data at different time points as the pandemic unfolds and at various stages of lockdown (e.g., after returning partially to work) to validate and broaden the current findings. The handwashing checklist should also be complemented alongside other measures, particularly assessments of adolescents’ social distancing from peers (which were irrelevant at the time of this study as the current sample was mandated to be at home in isolation) and mask-wearing habits. Notably, this preliminary investigation’s sampling method of an online survey was an unavoidable methodology considering the lockdown conditions; however, the generalization of the current findings may be limited as this sample represents a specific population who answers internet surveys. Self-reporting and post facto techniques are known to have weaknesses, such as response biases, selective memory, positive self-attribution, and exaggeration of motivations [112]. Thus, future similar studies should be carried out in different countries and investigate different settings.

## Figures and Tables

**Table 1:** Means, standard deviations, and ranges for study variables (*n* = 651).

	Self-Control	Hope	Positivity Ratio	Pandemic-Related Anxiety	Social Support	Handwashing	
						Before the Pandemic	During the Pandemic
Range	−30–+70	10–64	0.36–3.80	8–53	12–72	0–12	0–13
M	14.19	45.79	1.49	25.39	49.88	4.77	6.72
SD	18.32	9.88	0.61	8.80	12.54	2.62	2.63

**Table 2:** Estimated regression weights (*n* = 651).

Paths			Estimate	StandardError	CriticalRatio	*p*
Self-control	🡢	Social support	0.225	0.038	5.876	<0.001
Self-control	🡢	Hope	0.438	0.035	12.541	<0.001
Self-control	🡢	Mid-pandemic handwashing	0.104	0.031	3.356	<0.001
Self-control	🡢	Pandemic-related anxiety	0.178	0.040	4.462	<0.001
Self-control	🡢	Pre-pandemic handwashing	0.177	0.039	4.589	<0.001
Self-control	🡢	Positivity ratio	0.268	0.040	6.695	<0.001
Hope	🡢	Positivity ratio	0.225	0.040	5.576	<0.001
Social support	🡢	Positivity ratio	0.079	0.036	2.166	0.030
Social support	🡢	Hope	0.160	0.035	4.589	<0.001
Social support	🡢	Pandemic-related anxiety	0.164	0.037	4.390	<0.001
Positivity ratio	🡢	Pandemic-related anxiety	−0.395	0.040	−9.964	<0.001
Pandemic-related anxiety	🡢	Mid-pandemic handwashing	0.154	0.030	5.056	<0.001
Pre-pandemic handwashing	🡢	Mid-pandemic handwashing	0.575	0.031	18.601	<0.001

## Data Availability

The datasets generated during and analyzed during the current study are available from the corresponding author on reasonable request.

## Supplementary Materials

The following are available online at https://www.mdpi.com/article/10.3390/ijerph18126280/s1, Table S1: Pearson’s correlation for the ten Pandemic-related anxiety (PRA) constructs.
