# Supplementary material for: The Role of Adolescents’ Personal and Social Resources in Achieving Desired Emotional and Behavioral Outcomes during an Anxiety-Provoking Pandemic Outbreak
Source: Int J Environ Res Public Health. 2021 Jun 10;18(12):6280. doi: 10.3390/ijerph18126280 (PMC8296054; doi:10.3390/ijerph18126280)
Supplement: Supplementary file 1 [file ijerph-18-06280-s001.zip › ijerph-1217765-supplementary.pdf]

International Journal of
Environmental Research and Public Health

Supplementary Material

Table S1. Pearson's correlation for the ten Pandemic-related anxiety (PRA) constructs.

	**PRA 1**	**PRA 2**	**PRA 3**	**PRA 4**	**PRA 5**	**PRA 6**	**PRA 7**	**PRA 8**	**PRA 9**	**PRA 10**
**PRA 1**	1	.666**	.572**	.451**	.407**	.444**	.267**	.494**	.317**	.516**
**PRA 2**		1	.531**	.492**	.362**	.448**	.370**	.373**	.275**	.449**
**PRA 3**			1	.458**	.369**	.273**	.152**	.419**	.238**	.398**
**PRA 4**				1	.318**	.312**	.254**	.336**	.300**	.350**
**PRA 5**					1	.227**	.165**	.290**	.169**	.297**
**PRA 6**						1	.682**	.354**	.246**	.507**
**PRA 7**							1	.276**	.240**	.393**
**PRA 8**								1	.383**	.440**
**PRA 9**									1	.276**
**PRA 10**										1

**. Correlation is significant at the 0.01 level (2-tailed).
